# P36 Lupus enteritis - a rare manifestation of systemic lupus erythematosus

**DOI:** 10.1093/rap/rkae117.067

**Published:** 2024-11-05

**Authors:** Swapan Nagpal, Harleen Kaur Miglani

**Affiliations:** Sukh Sagar Hospital, Amritsar, India; Nijjar Scan and Diagnostic Centre, Amritsar, India

## Abstract

**Introduction:**

Lupus enteritis is a rare manifestation of systemic lupus erythematosus (SLE). The presentation of this uncommon entity may range from an outpatient complaining of nausea, vomiting, and mild abdominal pain to an acutely ill patient in the emergency room with a surgical abdomen. Enteritis may be the only manifestation of an SLE patient in a flare. A high clinical index of suspicion and a CT scan of the abdomen are invaluable in diagnosing this uncommon albeit serious phenomenon.

**Case description:**

A fourteen-year-old girl presented with abdominal pain, nausea, vomiting, loss of appetite and weight for the last month. She had pallor, and there was a large painless erythematous ulcer on the hard palate (which appeared to have formed by coalesced multiple small ulcers). Her abdomen was tender and was doughy on palpation. She had been diagnosed with systemic lupus erythematosus one year ago based on clinical features of malar rash, alopecia and investigations suggesting proteinuria, ANA (IF) with anti-dsDNA and anti-Sm positivity. She has been on hydroxychloroquine and corticosteroids since then. She was diagnosed with pulmonary tuberculosis two years ago and was treated with anti-tuberculosis therapy. Blood investigations revealed hemoglobin 9.6 g/dl, leucocyte count 5500/microlitres, platelet count 1,80,000/microlitres, ESR 130 mm/hr, CRP 7 mg/dl, serum creatinine 0.7 mg/dl, no proteinuria. The tuberculin skin test (TST) was negative, C3 and C4 levels were low. Suspecting lupus enteritis, but also considering a close differential of tuberculosis; a contrast-enhanced abdominal CT was done that revealed mild ascites; submucosal edema the loop of the terminal ileum with a “target”-like pattern enhancement of the wall; diffuse thickening and edema of the cecum, ascending colon, transverse colon, and descending colon. The mesenteric vascularity was accentuated, and the vasa recta were prominent (comb sign). Based on the CT findings, negative TST, and low complement levels, we arrived at a diagnosis of lupus enteritis causing a lupus flare. Intravenous corticosteroids (methylprednisolone 80 mg IV once daily for 5 days) were administered, followed by prednisolone at 1 mg/kg and azathioprine. Within a week of therapy, there was remarkable improvement. She started eating well and began to gain weight. Repeat abdominal CT (after 6 weeks) showed near-complete resolution of the edema and submucosal thickening in the small and large bowel.

**Discussion:**

SLE commonly affects the skin, kidneys, heart, lungs, and central nervous system. Gastrointestinal manifestations of the disease are not common (except oral ulcers). They are mostly attributable to the medications used in treatment, or to the associated autoimmune conditions, such as autoimmune hepatitis, inflammatory bowel disease, primary biliary cirrhosis, and celiac disease. The various GI manifestations of SLE include oral ulcers, hepatitis, autoimmune pancreatitis, protein-loss enteropathy, and lupus enteritis. Lupus enteritis is a rare manifestation of SLE that may have serious consequences like intestinal necrosis, obstruction, and perforation if not diagnosed and treated in time.

The most common clinical feature of lupus enteritis is abdominal pain, followed by nausea, vomiting, diarrhea, and fever. These symptoms may lead to a loss of appetite and weight. It may also present as an acute surgical abdomen. One must rule out appendicitis, peptic ulcer, acute pancreatitis, diverticulitis, pseudo-obstruction, intestinal perforation, and mesenteric ischemia, before making this rare diagnosis. In endemic countries like India, tuberculosis is a very close differential diagnosis.

An abdominal CT scan is considered the gold standard for diagnosis. The classical findings on a CT scan are bowel wall edema, abnormal bowel wall enhancement (target sign), engorgement of and increase in the number of mesenteric vessels (comb sign), and increased attenuation of mesenteric fat.

The treatment of lupus enteritis consists of moderate to high doses of corticosteroids and immunosuppressive agents including mycophenolate mofetil, cyclophosphamide, and azathioprine.

The first clue of a lupus flare was the presence of a large, painless ulcer on the hard palate. Given a history of pulmonary tuberculosis, we had a strong suspicion of abdominal tuberculosis. However, the typical CT findings, low complement levels, and a negative tuberculin skin test tilted the scale in favour of lupus enteritis. The exceptional response to corticosteroids confirmed our diagnosis.

**Key learning points:**

• Among the various gastrointestinal manifestations of SLE, lupus enteritis is a life-threatening entity, albeit uncommon. Thus, it deserves early identification and prompt management.

• Painless hard palate ulcers may denote a high-disease activity state in SLE.

• An abdominal CT scan is considered the gold standard for diagnosis of lupus enteritis. The classical findings on a CT scan are bowel wall edema, abnormal bowel wall enhancement (target sign), engorgement of and increase in the number of mesenteric vessels (comb sign), and increased attenuation of mesenteric fat.

• It is important to consider differential diagnoses including appendicitis, peptic ulcer, acute pancreatitis, diverticulitis, pseudo-obstruction, intestinal perforation, and mesenteric ischemia.

• In endemic countries like India, one must also consider the possibility of tuberculosis.

